# The peri-operative management of patients with hereditary angioedema requiring dental treatment: a case series

**DOI:** 10.1038/s41415-026-9788-2

**Published:** 2026-07-24

**Authors:** Naomi Murray, Andrew Geddis-Regan

**Affiliations:** 576751485913036696699 University Dental Hospital, Manchester University NHS Foundation Trust, Manchester, M15 6FH, United Kingdom; 176961213439030823139 Division of Dentistry, University of Manchester, Manchester, M13 9PL, United Kingdom

## Abstract

Hereditary angioedema is a rare genetic disorder causing recurrent, potentially life-threatening swelling episodes, often triggered by dental procedures. Swellings resemble anaphylaxis, but pathophysiology and therefore management is distinctly different. This case series presents the management of four patients, emphasising multidisciplinary planning and highlighting the importance of awareness among dental professionals.

## Key points

- Hereditary angioedema can lead to life-threatening laryngeal oedema which can be triggered by dental treatment.
- A multidisciplinary approach with the patient's immunology team is vital to ensuring safe patient care.
- Currently no dental guidelines exist to inform the management of these patients; hospital-based care for active interventions should be considered.

## Introduction

Angioedemas are swellings in the lower layers of the skin due to increased vascular permeability, and can grossly be classified as allergic (i.e., mast cell or immunoglobulin E-mediated) or non-allergic. Hereditary angioedema (HAE), also known as C1-esterase inhibitor (C1-INH) deficiency, is a type of non-allergic angioedema estimated to affect one in 50,000 people.^1^ As the name would suggest, HAE is a genetic disorder which follows an autosomal dominant pattern, with three main types existing. Types I and II are caused by variants in the SERPING1 gene which codes for the C1-inhibitor protein.^1^^,^^2^^,^^3^ Type I is the most common accounting for approximately 85% of cases and is characterised by a deficiency of C1-INH, whereas Type II, which accounts for approximately 15% of cases, presents with normal levels of C1-INH but reduced function.^1^^,^^2^^,^^4^ Type III is rare and presents with normal levels and function of C1-INH. It is thought to be oestrogen-dependent, with a variety of different mutations attributed to this type.^1^^,^^2^^,^^3^

The pathophysiology of HAE is complex: the C1-inhibitor protein has roles in the complement system, the Kinin-Kallikrein system, the coagulation cascade, and fibrinolytic system.^2^ The complement system forms part of the innate immunity; it is a defence system made up of over 30 proteins produced by the liver which act in a cascade reaction to promote phagocytosis, cytolysis and inflammation. C1-INH inhibits the complement system to prevent spontaneous activation; low levels or reduced function allow for unchecked activation of complement proteins, facilitating vascular permeability.^2^ However, bradykinin is understood to be the main mediator of HAE and responsible for many of the symptoms seen in HAE.^1^^,^^2^^,^^3^^,^^4^ This pro-inflammatory peptide functions in endothelial contraction, increases vessel permeability, and activates nociceptors.^1^ C1-INH inhibits plasma kallikrein, the enzyme which cleaves high-molecular-weight kininogens to form bradykinin. Therefore, in HAE plasma, kallikrein levels and consequently bradykinin levels are allowed to rise.^3^^,^^5^ It is worth noting angiotensin-converting-enzyme (ACE) lowers bradykinin levels, ergo ACE inhibitors, a class of frequently used antihypertensive drugs, have been associated with cases of angioedema (ACE inhibitor-induced angioedema).^6^

The disorder typically presents in childhood and gets worse around puberty.^1^^,^^7^ Symptoms include localised subcutaneous swellings involving the face and extremities.^1^^,^^8^ Swellings are hard, painful and non-pitting, with ill-defined margins and no urticarial rash or itching, distinguishing them from allergy/anaphylaxis.^8^ There may also be involvement of the gastrointestinal tract, which presents as accompanying abdominal symptoms, including pain and distension, nausea, vomiting, diarrhoea, or constipation.^1^^,^^2^^,^^3^^,^^8^ Involvement of the respiratory tract can present as potentially life-threatening laryngeal oedema if progression to asphyxiation occurs.^7^^,^^8^ The onset of swellings is usually over several hours; if left untreated, symptoms usually increase over two days and subside after two to five days.^1^ The frequency of attacks varies between individuals with precipitating factors including infections, psychological stress, menstruation, drugs (namely ACE inhibitors and oestrogens), trauma, surgery, and dental treatment.^1^^,^^2^^,^^4^^,^^6^

Diagnosis is based on clinical history and laboratory tests.^1^^,^^2^^,^^4^^,^^8^ Diagnostic delay or misdiagnosis is not uncommon owing to overlapping symptoms with more common conditions;^7^ therefore, care should be taken to exclude urticaria or an infective cause for swellings, with failure to respond to long-term antihistamine therapy being a key diagnostic indicator.^1^^,^^3^ Genetic testing can also be conducted; however, this cannot predict the disease severity or symptom frequency.^1^^,^^4^

Treatment can be divided broadly into on-demand for acute attacks and prophylaxis, with the latter including short-term prophylactic cover for known triggers such as surgery, and long-term prophylaxis to reduce frequency of attacks and overall disease burden.^1^^,^^3^^,^^4^^,^^9^ Currently, in the United Kingdom (UK), the appropriateness for long-term prophylactic medication is determined by attack frequency.^9^ On-demand treatment includes plasma-derived C1-INH products, recombinant C1-INH, or bradykinin B2 receptor antagonists.^3^^,^^4^ Patient education is key, with self-administration being crucial for timely and effective on-demand treatment.^4^ C1-INH products are given intravenously whereas bradykinin B2 receptor antagonists are given subcutaneously; patients and ideally a second ‘home therapy partner' should be therefore trained appropriately for self-administration.^4^ C1-INH products are recommended as first-line short-term prophylaxis. Fresh frozen plasma may also be used; however, this carries a greater risk as transmission of bloodborne viruses or allosensitisation may occur.^4^ Long-term prophylaxis may consist of C1-INH products, plasma kallikrein monoclonal antibody, plasma kallikrein inhibitors, or androgens.^1^^,^^3^^,^^4^ Tranexamic acid, an antifibrinolytic drug which dental professionals may be familiar with for its use in achieving haemostasis, may be used for prophylaxis but is not routinely recommended unless first-line treatment is not available and androgens are contraindicated.^4^ They act by inhibiting the conversion of plasminogen to plasmin, thereby reducing bradykinin production.^3^ Evidence supporting their efficacy remains limited,^4^ although some patients report finding them beneficial.^3^ Table 1 outlines on-demand and prophylactic treatments commonly used in the UK.

**Table 1:** Treatment for hereditary angioedema^1^^,^^3^^,^^4^

**Drug type**	**Mechanism of action**	**Administration route**	**Comments**
**On-demand treatment**			
Cinryze, Berinert	Plasma-derived human C1-INH replacement	Intravenous infusion	Additional infusion can be given if symptoms persist >1 hour. Vaccinations for hepatitis A and B are recommended
Ruconest	Recombinant C1-INH replacement (derived from rabbit milk)	Intravenous infusion	Additional infusion can be given if symptoms persist >2 hours. Contra-indicated with rabbit allergy
Icatibant	Selective bradykinin B_2_ receptor antagonist	Subcutaneous injection	Generally well tolerated
**Short-term prophylaxis**			
Cinryze, Berinert, Ruconest (plasma-derived or recombinant C1-INH as above)			
**Long-term prophylaxis**			
Androgens (Danazol, Oxandrolone, Stanozolol)	Induce hepatic synthesis of C1-INH	Oral (dose dependent on specific medication)	Contra-indicated in childhood, pregnancy, lactation, prostate cancer. Liver function and lipid profile requires monitoring
Berotralstat	Plasma kallikrein inhibitor	Oral, once daily	Liver function requires monitoring. Baseline echocardiogram as increased risk of QT prolongation
Lanadelumab	Monoclonal antibody directed against plasma kallikrein	Subcutaneous injection, initially every 2 weeks	Licenced for patients aged 12 or over. Manufacturer advises avoid in pregnancy
Cinryze	Plasma-derived human C1-INH replacement	Intravenous, every 3–4 days	Preferred during pregnancy and lactation
C-IHN, C1-esterase inhibitor			

As HAE is an inherited condition, multiple family members may be affected, and despite the availability of treatment, swelling attacks can be unpredictable. The disease burden and impact on quality of life therefore is significant, not only on the affected individual, but family and caregivers alike. Attacks can be disruptive of daily function, including requiring time off work or school.^10^ The impact on mental health is considerable, with fear of attacks limiting social engagement, and many patients have concerns about passing it onto their children.^10^ There is no doubt that patients would benefit from psychological support; however, the current public sector provision of services in the UK means this is generally difficult to access.^9^

As previously noted, dental treatment can trigger a swelling episode. Other procedures involving mechanical stimulation of the upper aerodigestive tract, such as intubation, may also trigger angioedema near the site of intervention.^4^ The likelihood and severity of such episodes can vary considerably, not only between patients but also within the same patient across different treatment episodes;^11^^,^^12^^,^^13^^,^^14^ therefore, the unpredictable nature of this condition can make the provision of dental care particularly challenging for both the patient and the clinician.

This case series details the management of four patients with hereditary angioedema who required dental treatment. As dental treatment can pose a major trigger for HAE attacks, the aim of this report is to raise awareness of this rare condition among dental professionals and to highlight the importance of careful treatment planning and appropriate onward referral based on the clinical setting.

## Cases

### Case 1

**Fig. 1:**
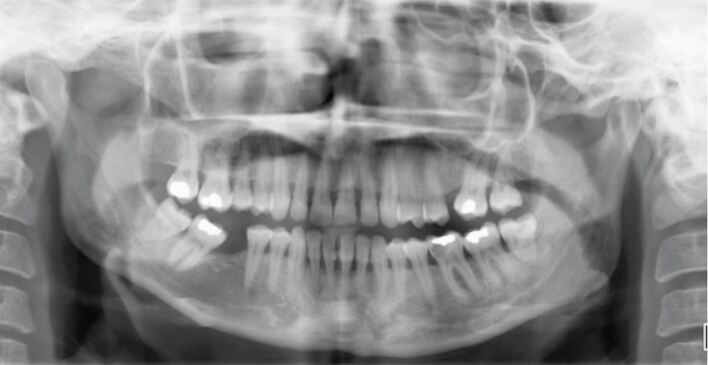
Orthopantomogram showing radiolucencies indicative of caries in 36 and 38

A woman in her forties with Type I HAE attended the Department of Special Care Dentistry for a new patient assessment. Her principal complaint was severe pain from the lower left quadrant. Following clinical and radiographic examination (Fig. 1) a diagnosis of irreversible pulpitis 36, caries 38, and generalised periodontitis was made. A plan was agreed with the patient to remove 36 and 38, and carry out an initial round of professional mechanical plaque removal in one further appointment. The patient's immunology team were contacted and informed of the dental treatment plan; they advised 4,200 units of Ruconest to be administered intravenously before the procedure, using the patient's own home supply.

**Fig. 2:**
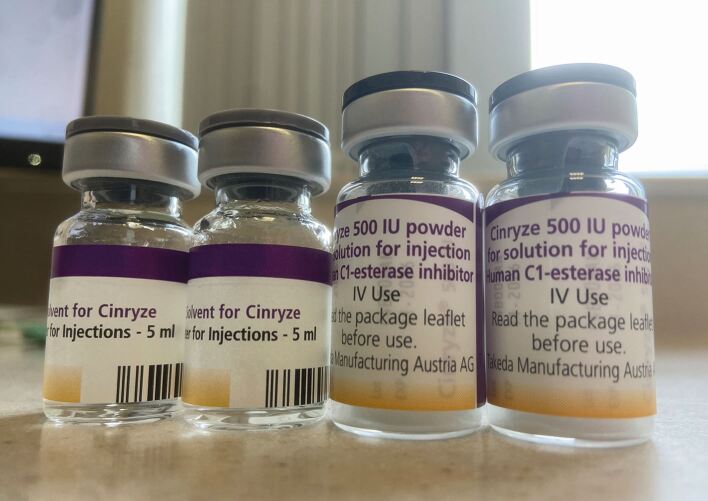
Cinryze vials of powder and solvent requiring reconstitution before administration

The following visit, the dental team administered 4,200 units Ruconest intravenously; 36 and 38 were removed under local anaesthetic without complication. The patient declined professional mechanical plaque removal and requested a further appointment to have this completed due to dental anxiety. The immunology team were contacted again for advice as to whether immunological cover was still required given the less invasive nature of the outstanding procedure. It was advised that the patient still required cover; however, due to a change in medication regimen, 1,000 units of Cinryze (Fig. 2) was recommended before dental treatment. Full-mouth supra- and subgingival professional mechanical plaque removal was provided at a subsequent visit following the administration of 1,000 units of Cinryze, once again administered intravenously by the dental team. No problems arose.

### Case 2

**Fig. 3:**
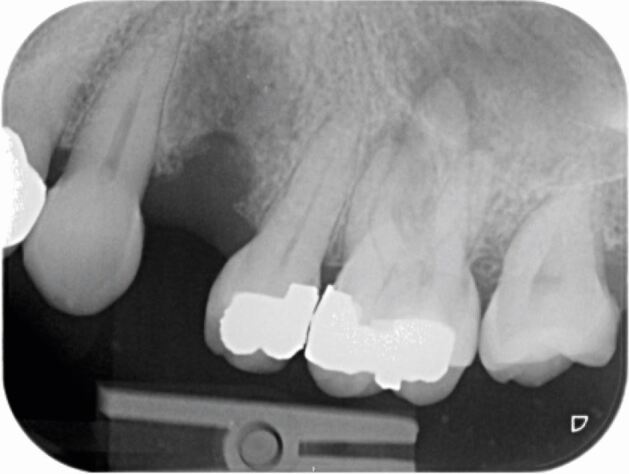
Periapical radiograph of the upper left first molar tooth showing apical pathology

This case involved a woman in her sixties with Type I HAE. On previous assessment, 26 was found to have an area of apical pathology on a periapical radiograph (see Fig. 3); however, the patient had declined treatment as the tooth was asymptomatic and she had an upcoming family wedding. She re-attended six months later reporting a history of swelling in the upper left quadrant and requested the tooth to be removed. Although the patient had previously received dental treatment under local anaesthetic, she expressed finding extractions difficult to cope with and requested sedation. Her immunology team advised 4,200 units of Ruconest before dental treatment, which was administered by the dental team intravenously. Removal of 26 was then carried out under intravenous sedation with midazolam without complication. The dental team ensured the patient had further supply of Ruconest at home should an attack occur post-operatively.

### Case 3

**Fig. 4:**
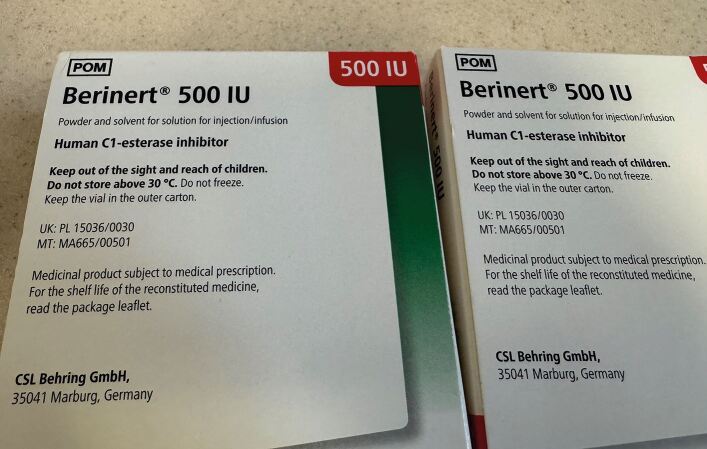
Berinert plasma-derived C1-INH replacement. Available in 500-, 1,500-, 2,000- and 3,000-unit powder and solvent formulation

An older woman attended for the removal of asymptomatic 47 under local anaesthetic due to unrestorable caries. Medical history included hypertension and stable Type I HAE. Following the initial assessment, advice was sought from the patient's immunology team who advised that for a simple single tooth extraction, pre-treatment cover may not be required but will reduce the risk of swelling, so 1,500 units of Berinert (Fig. 4) should be given within two hours of the procedure, and the patient should bring a second dose in case of post operative swelling. On the day of treatment, the dental team administered 1,500 units of Berinert intravenously via a cannula, and 47 was removed under local anaesthetic via inferior dental block without complication.

### Case 4

A teenage female patient with Type 1 HAE was referred to the Department of Special Care Dentistry by her immunology team due to wisdom tooth pain. The patient was taking long-term prophylaxis due to poorly controlled HAE with swelling episodes being reported twice weekly. She was started on Lanadelumab 300 mg subcutaneous injections every five weeks with a plan to receive Berinert 1,000 units if needed for breakthrough severe swellings. This drug regimen was then increased to seven weeks spacing as swelling control was achieved with no requirement for on-demand treatment.

**Fig. 5:**
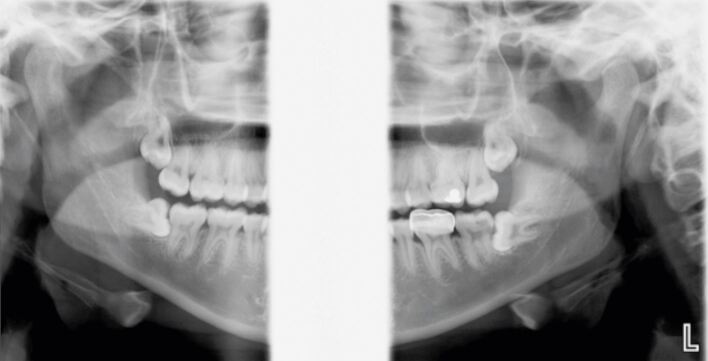
Case 4 sectional orthopantomogram showing horizontally impacted 48 and 38, and radiolucency indicative of caries in 37

The patient attended complaining of pain from the lower right wisdom tooth but no history of swelling or infection. On examination, the lower right wisdom tooth was partially erupted with no suppuration or swelling; all other wisdom teeth were unerupted. Radiographic examination revealed horizontal impaction of 38 and 48, and occlusal caries 37 (Fig. 5). The patient did not meet the National Institute for Health and Care Excellence guidelines for removal of wisdom teeth^15^ but required restoration of 37. The immunology team was consulted and indicated uncertainty regarding the full protective effect of the biologic therapy. They recommended administration of 1,500 units of Berinert if the procedure necessitated general anaesthesia, although did not feel this was strictly required. For procedures under local anaesthesia, it was advised to schedule the treatment during the week following the patient's Lanadelumab infusion, when her risk is presumed to be lowest, ensuring access to Berinert only if needed to minimise unnecessary use while maintaining safety.

Based on this advice, an appointment was scheduled to coincide with one week following the patient's Lanadelumab infusion. Restoration of 37 was completed uneventfully under local anaesthetic. A cannula was inserted pre-procedure should the need for quick venous access arise; however, Berinert was not required. The patient was followed-up with a telephone call two days after and confirmed she had not experienced any post-operative angioedema attacks.

## Discussion

This case series describes the management of four patients with HAE. The disease burden and impact on quality of life is variable between patients,^7^^,^^9^ with studies demonstrating patients with HAE may experience barriers to accessing dental care, such as avoidance due to fear of attacks or anxiety regarding refusal of treatment by the clinician; therefore, an awareness of this condition is imperative among dental professionals.^11^^,^^12^^,^^13^ Furthermore, emotional stress itself can trigger attacks, therefore dental anxiety can potentially worsen an acute attack.^16^ The patient in Case 2 was given intravenous sedation with midazolam, which is less restrictive, and considerably safer than general anaesthetic as intubation itself may induce swelling.^4^^,^^16^ The patient in Case 1 was offered sedation but declined and was instead treated under local anaesthetic with non-pharmacological dental behavioural support techniques including ‘stop signal' and ‘counting time'.^17^ The importance of anxiety control is underestimated, likely due to lack of literature; however, managing anxiety may help to prevent and/or reduce attacks.^16^^,^^18^ Moreover, patients should be informed that the sensations associated with local anaesthetic may resemble angioedema-related swelling, especially if block anaesthesia is administered, and should be reassured that this is a typical pharmacological effect rather than an indication of an attack.

For each case, the authors liaised with the patient's immunology team to ascertain the individualised risk of the proposed treatment, and therefore what, if any, immunological cover was required. It is recommended patients always carry on-demand medication with them and should be supplied with sufficient medication for on-demand treatment of at least two attacks.^4^ It is therefore advised that patients bring two doses of prophylactic medication with them to their appointment, one to be given pre-procedure, and a second should an attack arise. Early on-demand treatment provides a better response, so it is essential that the patients maintain an adequate supply.^4^ When planning dental procedures, the potential delay in accessing on-demand medication must be considered, as not all hospitals keep it readily available and it may be difficult to source quickly in the event of unexpected angioedema. Patients should be informed of the risk of attacks following the procedure, regardless of whether short-term prophylaxis has been administered. It is important to note that, unlike swelling associated with anaphylaxis, HAE does not respond to antihistamines, adrenaline, or glucocorticoids.^1^^,^^3^^,^^8^ Patients should therefore be aware of the most appropriate facility to seek emergency care, should the need arise, as specialist centres or a coordinated network of HAE specialists may exist.^9^

The dental team (who consisted of clinicians working in the Department of Special Care Dentistry) administered the prophylactic cover, as they were comfortable with gaining intravenous access and administering medications via this route. This approach to patient care eliminated the need for immunological cover to be provided by a separate team, thereby reducing the time and cost burden of attending multiple appointments at different locations for the patient. Alternatively, the patient or their ‘home therapy partner' may be amenable to administering the required cover.^4^ There is limited literature and no available guidelines to support dental professionals in the appropriate management of this patient group; however, considerations should be made regarding the appropriate care setting and who should administer the prophylaxis cover. Treatment modifications may need to be considered based on what is realistic and appropriate, as for other patients with complex risk requirements. Thus, it is of the authors' opinion, given that the administration of C1-INH products is typically done by patients themselves or immunology teams, that administration by dental teams in a hospital setting is not unreasonable.

All patients in this case series were treated in a hospital setting as this was deemed to be the most appropriate setting should issues arise, due to immediate access to medical assistance and those trained in advanced airway management. This was particularly pertinent for Case 4, where the patient had recently started a new long-term prophylaxis therapy, and the severity of dental treatment as a trigger had not yet been tested while the patient was receiving this new medication regimen. Management of these patients in a primary care setting may be suitable for those with well-controlled disease, a history of good tolerance to routine dental care, and the ability to self-administer prophylactic treatment, but prior consultation with their immunology team should confirm this. Though immunological cover largely mitigates risk, complex multi-stage invasive procedures over numerous visits may need very cautious consideration as to their appropriateness and feasibility, and referral to specialist dental services is reasonable should the required treatment exceed the clinician's scope of practice or competence.^19^

Attention to treatment planning should also be made with the goal of promoting more sustainable practices within the NHS. Immunological cover comes with a heavy cost implication, with the short-term prophylaxis provided for the patients in this case series ranging from £1,340 to £2,672 per dose.^20^ Case 4 demonstrates an example of sustainable practice, as the patient's immunology team recommended not to provide short-term prophylaxis but ensuring access if needed, reducing waste while maintaining safety.

Attempts should therefore be made to reduce the number of visits where possible. However, a holistic approach to patient care must always be taken, with consideration of patient wishes and preferences in relation to how much treatment is carried out per appointment. In this context, the importance of preventing dental disease cannot be overstated. Maintaining optimal oral hygiene and adhering to healthy dietary practices help prevent the development of odontogenic disease and its consequential impact on quality of life.^21^ Preventing dental disease from the outset can also reduce the need for dental treatment and hospital-based care with immunological cover, thereby minimising the risk of triggering a swelling episode and the associated burden on healthcare resources, respectively. In conclusion, dental education and the appropriate professional preventive interventions based on risk assessment is paramount.^22^

## Conclusion

HAE can lead to life-threatening laryngeal oedema. Despite the possibility of dental treatment triggering swelling attacks, patients with this condition are safe to treat as demonstrated by this case series. Liaison with the patient's immunological team before commencing dental treatment should be mandatory in all instances as patients present with variable regimes and risk profiles, and required prophylactic medications can be given by dentists if they are comfortable with intravenous access and drug administration. In summary, this case series highlights the importance of multidisciplinary collaboration and the need for a holistic approach to dental care to support streamlined and safe access to care.
